# Impact of the Total Number of Carotid Plaques on the Outcome of Ischemic Stroke Patients with Atrial Fibrillation

**DOI:** 10.3390/jcm8111897

**Published:** 2019-11-07

**Authors:** Hyungjong Park, Minho Han, Young Dae Kim, Joonsang Yoo, Hye Sun Lee, Jin Kyo Choi, Ji Hoe Heo, Hyo Suk Nam

**Affiliations:** 1 Department of Neurology, Yonsei University College of Medicine, Seoul 03722, Korea; hjpark209042@gmail.com (H.P.); UMSTHOL18@yuhs.ac (M.H.); neuro05@yuhs.ac (Y.D.K.); JKSNAIL85@yuhs.ac (J.K.C.); jhheo@yuhs.ac (J.H.H.); 2 Department of Neurology, Keimyung University School of Medicine, Daegu 42601, Korea; quarksea@gmail.com; 3 Biostatistics Collaboration Unit, Yonsei University College of Medicine, university, Seoul 03722, Korea; HSLEE1@yuhs.ac

**Keywords:** atrial fibrillation, cerebral infarction, carotid stenosis, ultrasonography, outcomes

## Abstract

Background: Atrial fibrillation (AF) shares several risk factors with atherosclerosis. We investigated the association between total carotid plaque number (TPN) and long-term prognosis in ischemic stroke patients with AF. Methods: A total of 392 ischemic stroke patients with AF who underwent carotid ultrasonography were enrolled. TPN was assessed using B-mode ultrasound. The patients were categorized into two groups according to best cutoff values for TPN (TPN ≤ 4 vs. TPN ≥ 5). The long-term risk of major adverse cardiovascular events (MACE) and mortality according to TPN was investigated using a Cox hazard model. Results: After a mean follow-up of 2.42 years, 113 patients (28.8%) had developed MACE and 88 patients (22.4%) had died. MACE occurred more frequently in the TPN ≥ 5 group than in the TPN ≤ 4 group (adjusted hazard ratio [HR], 1.50; 95% confidence interval [CI], 1.01–2.21; *p* < 0.05). Moreover, the TPN ≥ 5 group showed an increased risk of all-cause mortality (adjusted HR, 2.69; 95% CI, 1.40–5.17; *p* < 0.05). TPN along with maximal plaque thickness and intima media thickness showed improved prognostic utility when added to the variables of the CHAD_2_DS_2_-VASc score. Conclusion: TPN can predict the long-term outcome of ischemic stroke patients with AF. Adding TPN to the CHAD_2_DS_2_-VASc score increases the predictability of outcome after stroke.

## 1. Introduction

Atrial fibrillation (AF) is the most common cause of cardioembolic stroke and is associated with poor prognosis in survivors after ischemic stroke. AF was reported to increase the annual risk of cardiovascular events by 5-fold [1,2]. In efforts to prevent cardiovascular events due to AF, researchers have focused on the identification of patients at high risk of developing cardiovascular events [3]. Several studies have suggested that atherosclerosis is associated with both the development and the outcome of AF [4]. For example, among the components of the CHAD_2_DS_2_-VASc score, age, hypertension, diabetes, history of stroke/transient ischemic attack, and vascular disease are known to be the important risk factors for atherosclerosis [5,6].

Carotid atherosclerosis ≥ 50% in patients with AF is well known to be an independent risk factor for future ischemic stroke and vascular events [7,8,9]. However, the prognostic implication of carotid atherosclerosis < 50% is not well known. Carotid ultrasonography can easily detect mild carotid atherosclerosis through measurements of the carotid intima media thickness (IMT) and carotid plaque thickness [8,9,10]. However, little is known about the prognostic impact of the number of carotid plaques on the outcome of patients with AF. In this regard, we evaluated the association between the total carotid plaque number (TPN) and long-term prognosis in ischemic stroke patients with AF.

## 2. Materials and Methods

### 2.1. Study Population

This is a hospital-based observational study in ischemic stroke patients who were prospectively registered to a stroke registry from January 2007 to December 2013 in Severance Hospital, Seoul, South Korea. [11]. The registry enrolled consecutive patients with acute ischemic stroke within 7 days of onset. During admission, all patients were evaluated with brain magnetic resonance imaging and/or computed tomography, as well as cerebral angiography (magnetic resonance angiography, computed tomography angiography, or digital subtraction angiography). Systemic evaluation included 12-lead electrocardiography (ECG), chest radiography, standard blood tests, lipid profile, and continuous ECG monitoring during stay in the stroke unit. Specific evaluation for finding the cardioembolic source, such as transthoracic echocardiography, transesophageal echocardiography, and 24-h Holter monitoring was done.

The stroke subtypes according to the Trial of ORG 10172 in Acute Stroke Treatment (TOAST) classification [12] and the presence of angiographic abnormalities were prospectively determined using neuroradiologist reports and the consensus of stroke specialists in weekly stroke conferences, and prospectively entered into a computerized database.

This study was approved by the institutional review board of Severance Hospital, Yonsei University Health System, which waived the requirement for informed consent from patients owing to the retrospective nature of the analysis.

### 2.2. Clinical Variables

We collected data on demographics and risk factors of stroke including hypertension, diabetes, hyperlipidemia, coronary artery disease, peripheral artery disease, history of stroke, transient ischemic accident or thromboembolism, and smoking habit. Hypertension was defined as a systolic blood pressure of ≥ 140 mmHg or a diastolic blood pressure of ≥ 90 mmHg, or any history of anti-hypertensive agent use. Diabetes was defined as fasting glucose level ≥ 7.0 mmol/L, random blood glucose level ≥ 11.0 mmol/L, glycated hemoglobin ≥ 6.5%, or a history of oral hypoglycemic agent or insulin use. Hyperlipidemia was defined as serum total cholesterol ≥ 6.21 mmol/L, low-density lipoprotein cholesterol ≥ 4.14 mmol/L, or any history of use of lipid-lowering agents after a diagnosis of hyperlipidemia. AF was diagnosed on the basis of the findings of routine ECG, Holter monitoring, or continuous ECG monitoring on the current admission or before admission. Paroxysmal AF was also considered the presence of AF. Congestive heart failure was determined from the history of heart failure diagnosis, treatment with loop diuretics, and ejection fraction ≤35% on echocardiography. Coronary artery occlusive disease (CAOD) was defined as any history of unstable angina, myocardial infarction, and CAOD. Peripheral artery occlusive disease was defined as any history of a diagnosis of peripheral artery disease at any hospital regardless of the presence or absence of intervention or medication for peripheral artery disease. Patients were considered current smokers if they had smoked any cigarettes within 1 year before admission. Medication history including anti-coagulant, anti-platelet, anti-hypertensive, and lipid-lowering agent use was collected. Laboratory data were also obtained for complete blood count, lipid profile, blood urea nitrogen level, and creatinine level. The severity of stroke was determined using the National Institute Health Stroke Scale (NIHSS) score at admission.

### 2.3. Carotid Artery Assessment

Carotid artery plaques were assessed using B-mode ultrasound (iU22 ultrasound system; Philips, Bothell, WA, USA) with a 3-9-MHz multifrequency linear array transducer. All measurements were done in a semi-dark room by two trained ultrasonographers. Bilateral longitudinal and transverse images of the common carotid arteries (CCAs) and internal carotid arteries (ICAs) were always obtained and the presence of plaque was decided after comparison of longitudinal and transverse images. The IMT in the CCAs was defined as the distance of the interface between the lumen-intima and the media-adventitia. The far wall of the carotid artery was visualized bilaterally in the CCAs (20–50 mm proximal to the bifurcation of blood flow), carotid bulb (0–20 mm proximal to the bifurcation of blood flow), and internal and external carotid arteries (0–20 mm distal to the bifurcation of blood flow). At 20, 25, and 30 mm proximal to the bifurcation of blood flow, IMT was bilaterally measured at the far wall of the CCAs during end-diastole, and calculated as the mean value for each patient. According to the Mannheim criteria [13], carotid plaque was defined as a focal structure encroaching into the arterial lumen by at least 0.5 mm, > 50% of surrounding IMT values, or thickness ≥ 1.5 mm above the distance of the interface between the lumen-intima and the media-adventitia.

The thickness of each plaque in the carotid arteries in the whole scanned area was also bilaterally measured. The TPN was determined by simply counting (bilaterally) the number of plaques in proximal ICAs and CCAs. The best cutoff values for TPN were determined using the Contal and O’Quigley method, which calculates the maximum hazard ratio (HR) based on log-rank statistics [14].

### 2.4. Follow-Up and Outcomes

After discharge, each patient was followed up with regularly at 3 months, 1 year, and yearly thereafter. At each follow-up visit, medical information including occurrence of any cardiovascular events, newly detected vascular risk factors, lifestyle modification after stroke, and re-admission to another hospital was obtained via face-to-face interviews with neurologists or through clinical research associates in the outpatient clinic. When the patients missed a scheduled visit, we obtained the information from the patients or their proxy through a telephone interview with a structured questionnaire [15]. In addition, we also obtained mortality data based on death certificates from the Korean National Statistical Office (http://www.kostat.go.kr).

The primary end point was major adverse cardiovascular events (MACE; cardiovascular mortality, non-cardiovascular mortality, and occurrence of non-fatal stroke or myocardial infarction). Cardiovascular mortality was defined as any mortality due to stroke, myocardial infarction, other cardiac disease, or unobserved sudden death. The secondary outcome was all-cause mortality. The censoring date was December 31, 2013.

### 2.5. Statistical Analysis

The data were presented as mean ± standard deviation or medians (interquartile range [IQR]), as appropriate. Differences between the two groups were compared with the chi-square test, Fisher’s exact test, Student’s *t*-test, and the Mann–Whitney U-test, as appropriate. Survival analysis was conducted, and survival curves were plotted using Kaplan–Meier analysis. The difference of survival time between groups was analyzed using a log-rank test. To determine the independent predictor of MACE and all-cause mortality, Cox proportional hazard regression analysis was used, and HR and 95% confidence interval (CI) values were summarized. Cox proportional hazard regression analysis was conducted with adjustments for age, sex, initial NIHSS score, and variables with *p* < 0.1 in the univariate analysis.

To evaluate the added value of carotid plaque burden for the prognosis of ischemic stroke caused by AF, we constructed the model incorporating variables in the CHA_2_DS_2_-VAS_c_ score and other variables associated with carotid plaque burden such as IMT, maximal carotid plaque thickness, and TPN. We compared the following five models: (1) CHA_2_DS_2_-VAS_c_ score variables alone; (2) addition of IMT; (3) addition of maximal carotid plaque thickness; (4) addition of TPN; and (5) addition of IMT, maximal carotid plaque thickness, and TPN. For internal validation of the newly developed model, time-dependent receiver-operating characteristic curves and areas under the curve (AUCs) were determined based on Heagerty’s incident / dynamic AUCs during the median follow-up time [16]. A boot strapping method with 1000 re-samplings for calculating the 95% CI and the difference between the c-indices of each model was applied [17]. All tests were two-sided, and *p* < 0.05 was considered statistically significant. Statistical analysis was performed using R software, version 3.1.3 (R Foundation of Statistical Computing, Vienna, Austria).

## 3. Results

### 3.1. Patients’ Characteristics

**Figure 1:**
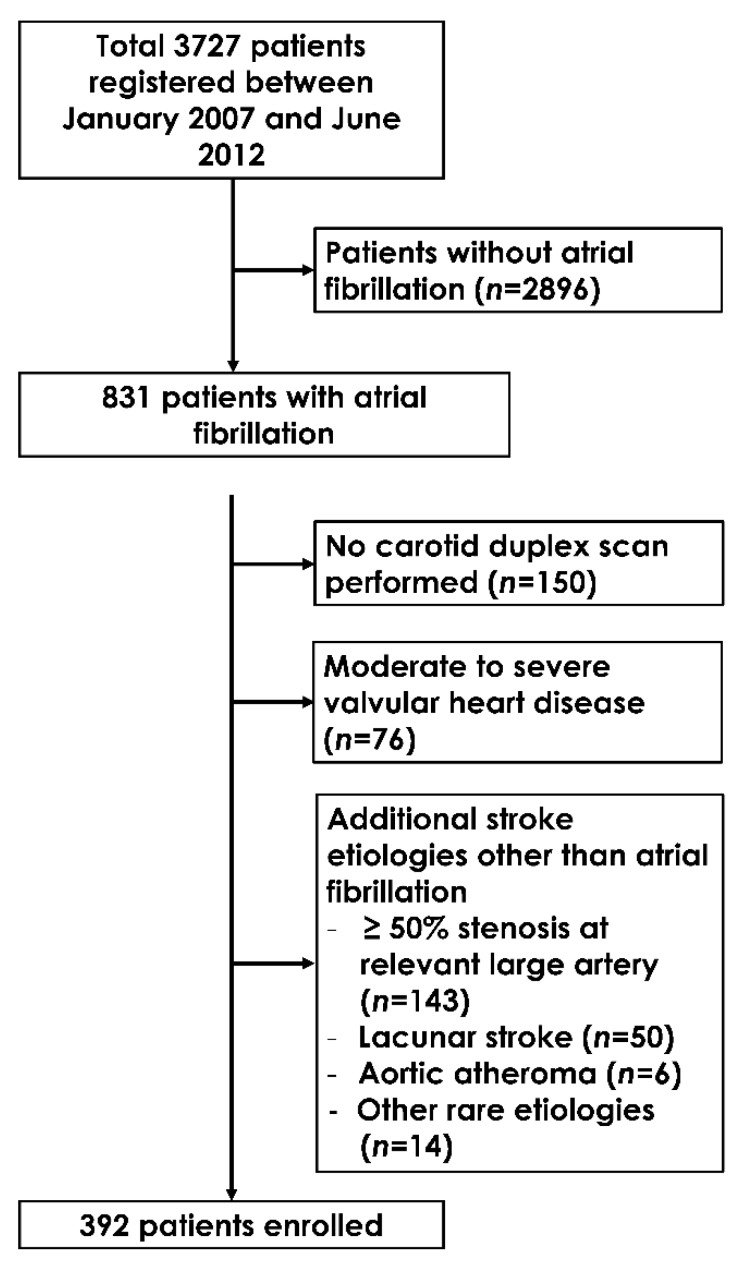
Flow sheet for study patients’ selection.

A total of 3727 consecutive ischemic stroke/transient ischemic attack patients were enrolled during the study period. After the exclusion of 2896 patients without AF, a total of 831 patients with AF remained. Among them, 150 patients without carotid ultrasonography and 76 patients with valvular heart disease were excluded. Patients who had > 50% stenosis in the intracranial or extracranial arteries (*n* = 143), complex aortic atheroma (≥ 4 mm or mobile atheroma) (*n* = 6), lacunar infarction (*n* = 50), and other rare etiologies (*n* = 14) according to the TOAST classification were also excluded. Finally, a total of 392 patients were analyzed (Figure 1).

The baseline characteristics of the enrolled patients are summarized in Table 1. The mean age of the total enrolled patients was 69.2 ± 10.3 years, and 225 (57.5%) patients were men. The median NIHSS score at admission was 5.5 (IQR 2–13). Before admission, 88 (22.4%) patients were taking oral anticoagulants. Carotid plaques were found in 343 (87.5%) patients. The median TPN was 3 (IQR 2–6). The median IMT and plaque thickness was 0.8 (IQR 0.7–0.9) and 2.1 (IQR 1.7–2.9), respectively. The inter-rater reliability based on the intraclass correlation coefficient (ICC) between ultrasonographers for carotid duplex sonography parameters was excellent, as follows: TPN (ICC: 0.983, *p* < 0.001), IMT (ICC: 0.966, *p* < 0.001), and maximal plaque thickness (ICC: 0.892, *p* = 0.001). In case of disagreement between ultrasonographers regarding parameters of the carotid duplex sonography, any disagreement was resolved by consensus. Following the Contal and O’Quigley method, the patients were categorized into two groups according to the best cutoff values for TPN (TPN ≤ 4 vs. TPN ≥ 5). The TPN ≤ 4 group consisted of 239 (71.0%) patients, and the TPN ≥ 5 group comprised 153 (39.0%) patients. Patients in the TPN ≥ 5 group were older and more likely to have hypertension, CAOD, statin use, or anti-hypertensive drug use. In addition, the TPN ≥ 5 group had higher IMT (0.9 ± 0.2 vs. 0.8 ± 0.2, *p* < 0.001) and larger maximal plaque thickness (3.0 ± 0.9 vs. 1.7 ± 1.1, *p* < 0.001) than the TPN ≤ 4 group.

### 3.2. Outcome

**Figure 2:**
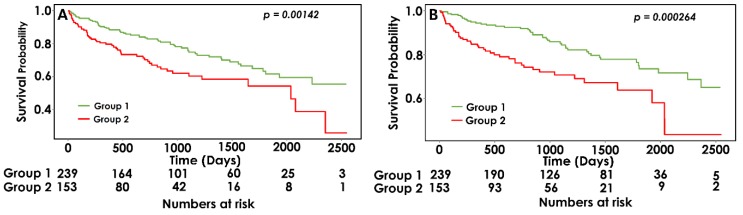
Kaplan–Meier analysis for (**A**) major adverse cardiovascular event (MACE); (**B**) all-cause mortality according to the total carotid plaque number (TPN).

The mean follow-up period was 2.42 ± 1.83 years. During the follow-up, a total of 113 (28.8%) MACE occurred in 60 (25.1%) patients of the TPN ≤ 4 group and in 53 (34.6%) patients of the TPN ≥ 5 group. In Kaplan–Meier analysis, the TPN ≥ 5 group showed a higher MACE rate than the TPN ≤ 4 group (log-rank test, *p*< 0.001) (Figure 2A). Multivariate Cox proportional regression analysis showed that the TPN ≥ 5 group had a significantly higher MACE rate than the TPN ≤ 4 group after adjusting for age, sex, and variables with *p* < 0.1 in univariate analysis (adjusted hazard ratio [HR], 1.50; 95% CI, 1.01–2.21; *p* < 0.05) (Table 2).

In terms of all-cause mortality, 88 (22.4%) patients had died during the follow up period. In Kaplan–Meier curve analysis, the TPN ≥ 5 group showed a higher mortality rate than the TPN ≤ 4 group (log-rank test, *p* < 0.001) (Figure 2B). In multivariate Cox proportional regression analysis after adjusting for age, sex, and variables with *p* < 0.10 in univariate analysis, patients in the TPN ≥ 5 group showed an increased risk of all-cause mortality (adjusted HR, 2.69; 95% CI, 1.40–5.17; *p* < 0.05) compared with patients in the TPN ≤ 4 group (Table 2).

### 3.3. Prognostic Utility of Carotid Plaque Burden on Ischemic Stroke Caused by AF

During the median follow-up period, the c-indices of Heagerty’s incident/dynamic AUC of each model were calculated (Table 3 and Supplementary Figure S1). The baseline model consisted of age, sex, congestive heart failure, diabetes mellitus, CAOD, and peripheral artery occlusive disease, which are the same variables of the CHAD_2_DS_2_-VASc score. The c-index for the baseline model was 0.651 (95% CI, 0.605–0.705) in MACE and 0.712 (95% CI, 0.658–0.766) in all-cause mortality. In model 5, including of all parameters of carotid plaque burden including TPN, maximal plaque thickness, and IMT improved prognostic utility that with the CHAD_2_DS_2_-VASc score alone in MACE (c-index, 0.686, 95% CI, 0.638–0.737, *p* = 0.045) and all-cause mortality (c-index, 0.734, 95% CI (0.686–0.786, *p* = 0.025). 

## 4. Discussion

The present study revealed that carotid plaque burden of < 50% carotid stenosis was a strong prognostic marker in patients with AF. Among the parameters of carotid plaque burden, TPN is easily counted during carotid ultrasonography examination. It showed an impact on the outcome of ischemic stroke patients with AF. Moreover, the carotid plaque burden improved the predictive value of the CHAD_2_DS_2_-VASc score in predicting cardiovascular events and mortality in ischemic stroke patients with AF. 

AF is the most common cause of cardioembolic stroke. Patients with AF had markedly reduced survival compared with those without AF. In the Framingham Heart Study, the risk factor-adjusted odds ratio for death was 1.5 and 1.9 in men and women, respectively [18]. Patients with AF frequently have concomitant cerebral atherosclerosis (20–50% of cases) [19,20]. It is well known that atherosclerosis is a systemic disorder that plays an important role in the prognosis of patients with AF [4]. It can be assumed that patients with AF are more likely to have additional atherosclerotic burden and may have poor prognosis. We previously reported that patients who have both large artery atherosclerosis (>50% atherosclerotic stenosis in the relevant artery) and cardioembolism showed higher cardiovascular mortality than patients with a single cause of either large artery atherosclerosis or cardioembolism [21]. Thus, it can be inferred that concomitant carotid atherosclerosis with AF is associated with the development of cardiovascular events despite the presence of < 50% stenosis.

To date, little is known about the impact of < 50% atherosclerotic stenosis of the carotid artery on the outcome of ischemic stroke patients with AF. The presence of large artery atherosclerosis can be screened using luminography including computed tomography angiography, magnetic resonance angiography, or digital subtraction angiography. However, arterial wall changes including small plaques or increased IMT in the carotid artery cannot be detected using luminography. Carotid ultrasonography is a noninvasive imaging examination that can easily and accurately evaluate carotid plaques and IMT in the arterial lumen. 

We found that the TPN ≥ 5 group had a 1.5-fold higher MACE rate than the TPN ≤ 4 group after adjustments. Moreover, considering all parameters of carotid plaque burden, including TPN, maximal plaque thickness, and IMT, contributed to the improvement of the risk stratification of ischemic stroke patients with AF over that with the CHAD_2_DS_2_-VASc risk score alone. The components of the CHAD_2_DS_2_-VASc score are clinical variables including old age, hypertension, diabetes, and vascular disease. These variables are also well-known risk factors for atherosclerosis [6]. Therefore, adding the carotid plaque burden to the model improves the risk prediction.

In line with our findings, cohort studies including non-stroke patients also reported similar results. In ARAPACIS (Atrial Fibrillation Registry for Ankle-brachial Index Prevalence Assessment: Collaborative Italian Study), a prospective nationwide observational cohort study in patients with non-valvular AF, the investigators reported that carotid plaque detection improves the predictive value of the CHAD_2_DS_2_-VASc score in patients with AF [22]. The ARIC (Atherosclerosis Risk in Communities) study investigators also reported that carotid IMT and the presence of carotid plaque are associated with an increased risk of ischemic stroke in patients with AF. The addition of carotid IMT and carotid plaque to the model provided an incremental predictive value for the risk of stroke over the CHAD_2_DS_2_-VASc score alone in adults with AF who had no prior ischemic stroke. Although we reached similar findings, a difference of the present study from the two cohort studies is that we enrolled only ischemic stroke patients with AF. Another difference is that we adopted TPN because this variable can be easily and acutely measured on routine carotid ultrasonography [10].

Currently, the method for the secondary prevention of ischemic stroke caused by AF is anticoagulation with a vitamin K antagonist or a direct oral anticoagulant (DOAC) [23]. However, vitamin K antagonists can prevent only 67% of future ischemic stroke events and DOAC did not show superiority over vitamin K antagonists [24,25]. Identification of high-risk patients for future events despite anticoagulation treatment is important. Carotid atherosclerosis and atherosclerotic burden can be easily detected using carotid duplex ultrasonography. Although TPN is less accurate and operator-dependent method than quantification measurement of carotid plaque such as total plaque area [26,27], TPN can be easily counted and may be helpful in identifying high risk patients in daily clinical practice. Our study has several limitations. First, unstable plaque morphology and hypoechoic plaque are associated with an increased risk of ischemic stroke; however, we did not analyze the characteristics of individual plaques. Nevertheless, unstable carotid plaque is known to be prevalent in advanced carotid atherosclerosis, and our study did not include patients with >50% stenosis in an intracranial or extracranial artery. Thus, the influence of the morphologic feature of carotid plaques may be little. Second, carotid duplex ultrasonography was conducted by two ultrasonographers; however, the measurement agreement between them was high. Third, potential selection bias may exist. To minimize selection bias, we recruited consecutive ischemic stroke patients with AF.

## 5. Conclusions

In conclusion, TPN is an important risk predictor in ischemic stroke patients with AF. In addition, considering all parameters of carotid plaque burden including TPN, maximal plaque thickness, and IMT may contribute to improving the risk prediction in ischemic stroke patients with AF, compared with the prediction with the clinical variables of CHAD_2_DS_2_-VASc score alone. These findings suggest that carotid ultrasonography may be useful in reclassifying these patients.

## Figures and Tables

**Table 1:** Clinical characteristics of study patients according to the total carotid plaque number (TPN).

	TPN ≤ 4 (*N* = 239)	TPN ≥ 5 (*N* = 153)	*P* Value
**Demographics**			
Age, years	66.4 ± 10.5	73.5 ± 8.4	< 0.001
Sex, men	134 (56.1)	91 (59.5)	0.575
Initial NIHSS score	5 (2–13)	6 (2–13)	0.639
**Risk factors**			
Hypertension	155 (64.9)	126 (82.4)	< 0.001
Diabetes mellitus	53 (22.2)	46 (30.1)	0.102
Smoking	38 (15.9)	23 (15.0)	0.930
Hyperlipidemia	38 (15.9)	37 (24.2)	0.057
PAOD	5 (2.1)	7 (4.6)	0.275
CAOD	47 (19.7)	50 (32.7)	0.005
CHF	33 (13.8)	21 (13.7)	1.000
**Laboratory findings**			
Hemoglobin, g/dL	14.1 ± 2.1	13.5 ± 1.5	0.001
White blood cell, × 10^9^/L	8202.7 ± 2827.0	7830.1 ± 2939.5	0.211
Platelet, × 10^9^/L	225.6 ± 69.9	224.6 ± 70.0	0.885
Blood urea nitrogen, mmol/L	17.4 ± 6.3	18.8 ± 9.6	0.190
Creatinine, µmol/L	1.0 ± 0.8	1.3 ± 1.6	0.128
Total cholesterol, mmol/L	170.8 ± 35.8	167.2 ± 39.4	0.361
Triglyceride, mmol/L	96.2 ± 50.2	89.5 ± 45.3	0.184
HDL-cholesterol, mmol/L	45.0 ± 11.5	44.8 ± 11.9	0.865
LDL-cholesterol, mmol/L	106.5 ± 32.3	103.6 ± 35.7	0.399
**Premorbid medication**			
Antiplatelet agent	98 (41.0)	76 (49.7)	0.114
Anticoagulants	60 (25.1)	28 (18.3)	0.147
Statin	36 (15.1)	44 (28.8)	0.002
Antihypertensive agent	93 (38.9)	81 (52.9)	0.009
**Carotid duplex measurement**			
IMT, mm	0.8 ± 0.2	0.9 ± 0.2	< 0.001
Maximal plaque thickness, mm	1.7 ± 1.1	3.0 ± 0.9	< 0.001
Total plaque number, n	2 (1–3)	7 (5–10.5)	< 0.001

Data are shown as n (%), mean ± SD, or median (IQR). SD, standard deviation; IQR, interquartile range; NIHSS, National Institute of Health Stroke Scale; PAOD, peripheral artery occlusive disease; CAOD, coronary artery occlusive disease; CHF, congestive heart failure;; HDL, high density lipoprotein; LDL, low density lipoprotein; IMT, intimal medial thickness.

**Table 2:** Unadjusted and adjusted hazard ratio for MACE and all-cause mortality according to the total carotid number of plaque.

	MACE				All-Cause Mortality			
	Univariate Analysis		Multivariate Analysis		Univariate Analysis		Multivariate Analysis	
	HR (95% CI)	*P* Value	HR (95% CI)	*P* Value	HR (95% CI)	*P* Value	HR (95% CI)	*P* Value
**Demographics**								
Age	1.05 (1.03–1.07)	0.000	1.04 (1.01–1.06)	0.002	1.07 (1.05–1.10)	0.000	1.05 (1.02–1.08)	0.003
Sex	0.66 (0.45–0.95)	0.027	1.03 (0.67–1.59)	0.887	0.66 (0.43–1.00)	0.051	0.95 (0.49–1.84)	0.874
Initial NIHSS score	1.06 (1.03–1.08)	0.000	1.05 (1.02–1.08)	0.001	1.07 (1.04–1.00)	0.446	1.07 (1.03–1.11)	0.000
**Risk factors**								
Hypertension	1.32 (0.86–2.04)	0.208			1.21 (0.74–1.96)	0.446		
Diabetes mellitus	1.02 (0.66–1.56)	0.933			1.10 (0.68–1.78)	0.106		
Smoking	1.18 (0.72–1.93)	0.512			1.16 (0.66–2.02)	0.603		
PAOD	1.70 (0.69–4.18)	0.247			2.68 (1.08–6.65)	0.033	1.25 (0.36–4.41)	0.727
CAOD	1.51 (1.01–2.26)	0.046	1.14 (0.75–1.75)	0.532	1.74 (1.11–2.73)	0.016	1.18 (0.64–2.16)	0.588
CHF	1.66(1.05–2.61)	0.029	1.22 (0.76–1.97)	0.411	2.31 (1.43–3.72)	0.001	1.72 (0.83–3.57)	0.148
**Laboratory findings**								
Hemoglobin	0.88 (0.80–0.95)	0.002	0.91 (0.82–1.01)	0.909	0.86 (0.79–0.94)	0.001	0.94 (0.79–1.11)	0.466
White blood cell	1.00 (1.00–1.00)	0.831			1.00 (1.00–1.00)	0.836		
Platelet	1.00 (1.00–1.00)	0.361			1.00 (0.99–1.00)	0.040	1.00 (0.99–1.00)	0.998
BUN	1.02 (0.99–1.05)	0.157			1.03 (1.01–1.06)	0.018	1.01 (0.98–1.05)	0.590
Creatinine	1.05 (0.88–1.24)	0.600			1.08 (0.91–1.28)	0.371		
Total cholesterol	1.00 (0.99–1.00)	0.089			0.99 (0.99–1.00)	0.067		
Triglyceride	1.00 (1.00–1.00)	0.929	1.00 (0.99–1.01)	0.184	1.00 (1.00–1.00)	0.974		
HDL–cholesterol	1.00 (0.98–1.01)	0.844			1.00 (0.98–1.02)	0.987		
LDL–cholesterol	0.99 (0.99–1.00)	0.071	1.00 (0.99–1.00)	0.234	0.99 (0.99–1.00)	0.051		
**Premorbid medication**								
Antiplatelet agent	1.03 (0.71–1.49)	0.867			1.02 (0.67–1.56)	0.916		
Anticoagulants	0.78 (0.49–1.23)	0.288			0.83(0.50–1.40)	0.494		
Statin	1.41 (0.92–2.16)	0.117			1.28 (0.78–2.11)	0.330		
Antihypertensive agent	1.45 (0.99–2.13)	0.056			1.75 (1.13–2.70)	0.011	1.73 (0.89–3.38)	0.106
Total plaque number								
TPN ≤ 4 (reference)	1		1		1		1	
TPN ≥ 5	1.82 (1.25–2.64)	0.002	1.50 (1.01–2.21)	0.044	2.16 (1.41–3.29)	< 0.001	2.69 (1.40–5.17)	0.003

MACE, major adverse cardiovascular events; HR, hazard ratio; CI, confidential interval; National Institute of Health Stroke Scale; PAOD, peripheral artery occlusive disease; CAOD, coronary artery occlusive disease; CHF, congestive heart failure; BUN, blood urea nitrogen; HDL, high density lipoprotein; LDL, low density lipoprotein; IMT, intimal medial thickness.

**Table 3:** C-indices of Heagerty’s incident/dynamic AUC for predicting MACE and all-cause mortality

	MACE			All–Cause Mortality		
	c–Index (95% CI)	Difference	*P*–Value	c–Index	Difference	*P*–Value
Model 1 ^⁕^	0.651 (0.605–0.705)	Reference		0.696 (0.647–0.753)	Reference	
Model 2 ^†^	0.661 (0.613–0.714)	0.010 (−0.005–0.033)	0.267	0.712 (0.658–0.766)	0.016 (−0.005–0.046)	0.218
Model 3 ^‡^	0.672 (0.626–0.726)	0.020 (0.001–0.049)	0.214	0.716 (0.670–0.769)	0.019 (0.001–0.045)	0.113
Model 4 ^§^	0.657 (0.609–0.710)	0.006 (0–0.022)	0.317	0.701 (0.651–0.756)	0.005 (−0.001–0.021)	0.405
Model 5 ^‖^	0.686 (0.638–0.737)	0.034 (0.006–0.071)	0.045	0.734 (0.686–0.786)	0.038 (0.006–0.075)	0.025

AUC, area under the curve; MACE, major adverse cardiovascular events; CI, confidence interval. ⁕ Model 1: CHA_2_DS_2_-VAS_c_ variables (age, sex, hypertension, diabetes mellitus, congestive heart failure, coronary artery occlusive disease, peripheral artery occlusive disease) † Model 2: Model 1 plus carotid intima medial thickness; ‡ Model 3: Model 1 plus total number of plaque; § Model 4: Model 1 plus maximal thickness of plaque; ‖ Model 5: Model 1 plus carotid intima medial thickness plus maximal plaque thickness plus total number of plaque.

## Supplementary Materials

The following are available online at https://www.mdpi.com/2077-0383/8/11/1897/s1, Figure S1. The c-indices of Heagerty’s incident/dynamic AUC of each model (**A**) major adverse cardiovascular event (MACE); (**B**) all-cause mortality.
